# ILK Induces Cardiomyogenesis in the Human Heart

**DOI:** 10.1371/journal.pone.0037802

**Published:** 2012-05-29

**Authors:** Alexandra Traister, Shabana Aafaqi, Stephane Masse, Xiaojing Dai, Mark Li, Aleksander Hinek, Kumaraswamy Nanthakumar, Gregory Hannigan, John G. Coles

**Affiliations:** 1 Division of Cardiovascular Research, Hospital for Sick Children, Toronto, Canada; 2 University Health Network, University of Toronto, Toronto, Canada; 3 Cell Adhesion Signaling Laboratory, Monash Institute of Medical Research, Monash University, Melbourne, Australia; University of Bristol, United Kingdom

## Abstract

**Background:**

Integrin-linked kinase (ILK) is a widely conserved serine/threonine kinase that regulates diverse signal transduction pathways implicated in cardiac hypertrophy and contractility. In this study we explored whether experimental overexpression of ILK would up-regulate morphogenesis in the human fetal heart.

**Methodology/Principal Findings:**

Primary cultures of human fetal myocardial cells (19–22 weeks gestation) yielded scattered aggregates of cardioblasts positive for the early cardiac lineage marker nk×2.5 and containing nascent sarcomeres. Cardiac cells in colonies uniformly expressed the gap junction protein connexin 43 (C×43) and displayed a spectrum of differentiation with only a subset of cells exhibiting the late cardiomyogenic marker troponin T (cTnT) and evidence of electrical excitability. Adenovirus-mediated overexpression of ILK potently increased the number of new aggregates of primitive cardioblasts (p<0.001). The number of cardioblast colonies was significantly decreased (p<0.05) when ILK expression was knocked down with ILK targeted siRNA. Interestingly, overexpression of the activation resistant ILK mutant (ILK*^R211A^*) resulted in much greater increase in the number of new cell aggregates as compared to overexpression of wild-type ILK (ILK*^WT^)*. The cardiomyogenic effects of ILK*^R211A^* and ILK*^WT^* were accompanied by concurrent activation of β-catenin (p<0.001) and increase expression of progenitor cell marker islet-1, which was also observed in lysates of transgenic mice with cardiac-specific over-expression of ILK*^R211A^* and ILK*^WT^.* Finally, endogenous ILK expression was shown to increase in concert with those of cardiomyogenic markers during directed cardiomyogenic differentiation in human embryonic stem cells (hESCs).

**Conclusions/Significance:**

In the human fetal heart ILK activation is instructive to the specification of mesodermal precursor cells towards a cardiomyogenic lineage. Induction of cardiomyogenesis by ILK overexpression bypasses the requirement of proximal PI3K activation for transduction of growth factor- and β1-integrin-mediated differentiation signals. Altogether, our data indicate that ILK represents a novel regulatory checkpoint during human cardiomyogenesis.

## Introduction

Integrin-linked kinase (ILK) is a multidomain integrin adaptor protein that possesses widely conserved structural and signal transduction functions [1], [2]. ILK binds to cytoplasmic domains of ß1-, ß2-, and ß3-integrin subunits and nucleates a supramolecular complex at the site of focal adhesions that connects to the actin cytoskeleton, thereby linking the extracellular matrix to the cytoskeleton in a manner essential for bidirectional force transduction [2]. Adaptor complexes centered around ILK comprise a signaling platform that, in response to distinct signal inputs from integrins and growth factor receptor tyrosine kinases, activates signaling pathways regulating growth, survival, cell cycle progression, epithelial-mesenchymal transition, and cellular differentiation [1], [3].

In the postnatal heart, ILK serves dual function as a mechanoreceptor and as a nodal regulator of adaptive, prohypertrophic signaling [4]–[6]. ILK-deficient mice die early during embryonic development owing to defects in epiblast polarization with an abnormal distribution of F-actin [7]. Specific localization of ILK to costameric and Z-disc structures implies a functional role in the integration of cardiac mechanoreception and contractility [8]. Disruption of ILK kinase activity results in heart failure phenotype in zebrafish that is dependent upon ILK-mediated vascular endothelial growth factor signaling (VEGF) [9]. Conditional ILK deletion in the mouse heart causes spontaneous dilated cardiomyopathy and sudden death at 6 to 12 weeks of age [10], suggesting an important and distinct role of ILK during vertebrate cardiac morphogenesis.

ILK activation by growth factor stimulation is normally regulated in a phosphoinositide 3-kinase (PI3K)-dependent manner involving activation of ILK by phosphatidylinositol (3,4,5)-trisphosphate (PIP3), which interacts with the central pleckstrin homology (PH)-like domain of ILK [11]. ILK signaling induces downstream phosphorylation of Akt/PKB on Ser473 and glycogen synthase-3β (GSK-3β) on Ser9, providing a molecular basis for its prosurvival, prohypertrophic effects [4], [5], [10]. Interestingly, the ILK gene contains hypoxia responsive elements and upon exposure to hypoxia, activates endothelial cell (EC) expression of hypoxia inducible factor 1-α (HIF1-α) and VEGF; in turn, receptor tyrosine kinase activation by VEGF stimulates HIF-1α in an amplification loop involving PI3K and ILK activation [12]. ILK was revealed as an upstream regulator of the EC hypoxic stress response that controls the recruitment of endothelial progenitor cells to ischemic tissue [13].

ILK regulates the Wnt signaling pathway to stimulate β-catenin/T cell factor (Tcf) transcriptional activity through negative regulation of GSK-3β [3]. Chemical inhibitors of GSK-3β and activation of β-catenin promote expansion of embryonic and postnatal Islet-1 *(Isl1*) cardiac progenitor cells [14]. Tcf3 acts as a cell-intrinsic inhibitor of pluripotent cell self-renewal through repressive binding to the Oct4 promoter, so that ILK-mediated activation of the Tcf3 transcriptional complex would be predicted to favor cellular differentiation [15], [16]. Activation of the Wnt/β-catenin pathway promotes cardiogenesis in early phase mouse ES cells [17]. Together, these data support the hypothesis that ILK pathway activation promotes cardiomyogenesis in susceptible precardiomyogenic progenitor cells.

The results of this study identify ILK as a novel cardiotropic factor that promotes recruitment of human fetal heart cells to a cardiomyogenic fate.

## Results

### Adenoviral-mediated Overexpression of ILK in Cultures of Cells Isolated from Human Fetal Myocardium Enhances the Formation of Aggregates of Cardioblasts

**Figure 1:**
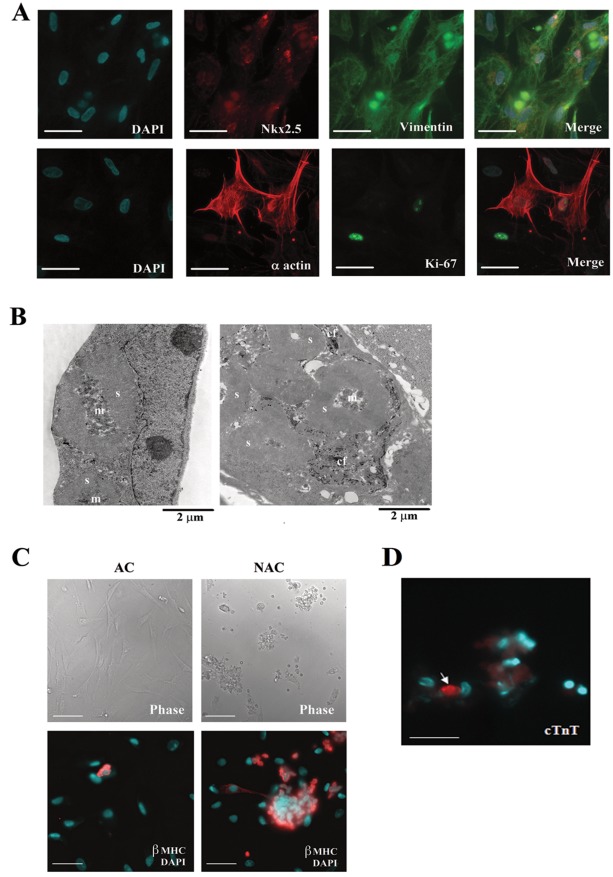
Cellular phenotypes in primary cultures of human fetal myocardium. (A) Cells freshly isolated from 22 week fetal myocardium were cultured for 2 days and then immunostained with anti-nk×2.5 (red) and anti-vimentin (green) (top panel) or with anti α-actin (red) and anti ki-67 (green) (bottom panel) antibodies to determine the percentage of the cells with cardiomyocyte or fibroblast phenotypes. Nuclei were marked with DAPI staining (blue). Scale bar, 30 µm. (B) Transmission electron microscopy showing that cultures of cells freshly isolated from human fetal myocardium at day 2 contain primitive cardioblasts with nascent sarcomeres (s) and mitochondrial clusters (m) (left) and cells with the transitional features containing both nascent sarcomeres and deep invaginations containing collagen fibers (cf) (right). (C) Only a subset of cardioblasts expressed cardiac troponin T (cTnT). (D) Phase contrast images (upper panel) and fluorescent images (lower panel) showing the adherent (AC) and non-adherent (NAC) cells 2 days after isolation. Immunostaining with anti-β-MHC antibody demonstrates that non-adherent clusters consist mainly of β-MHC positive cardioblasts. Scale bar, 80 µm (for phase) and 25 µm (for fluorescent imaging).

Initial immuno-histochemical screening demonstrated that the majority of cells isolated from human fetal myocardium (19–22 weeks gestation) were positive for vimentin, a specific maker for mesenchymal cells that is also expressed in fibroblasts, endothelial cells and myocytes at very early differentiation stage [18]. Approximately 35% of cells were positive for the early cardiac lineage marker nk×2.5, and ∼20% of cells were positive for muscle marker α-actin and cardiomyocyte-specific sarcomeric protein ß-myosin heavy chain (ß-MHC) and were Ki-67 positive (Figure 1A). Less than 5% of isolated cells demonstrated the presence of smooth muscle cell α-actin (data not shown). Electron microscopy (EM) showed that cardioblasts contained nascent sacromeric structures (Figure 1B). Many of these cells exhibited with a tight connection with extracellular collagen fibers (in some cases filling their invaginations) that were likely produced before their cardiomyogenic commitment (Figure 1B).

Freshly isolated cells were pre-incubated for 2 h and then the non-adherent fraction was separated from the adherent fraction of myocardial cells. Characterization of cells was performed within 7 days, with or without infection, following separation of cell fractions. We show by immunostaining and by EM that non-adherent cells were enriched with uniform aggregates of immature ß-MHC-positive cardioblasts (Figure 1C). These cellular aggregates were identical to those present among unfractioned cells and contain poorly organized sarcomeres adjacent to abundant clusters of mitochondria (Figure 1B). Only a small subset of cardioblasts expressed the late sarcomeric marker cardiac troponin T (cTnT) (Figure 1D). The fraction of initially floating and aggregating cells attached to the bottom of the culture dishes within 24 h of incubation. In contrast, the initially adherent cells were uniformly spread and did not aggregate (Figure 1C). This phenotypically diversified fraction of adherent cells contained fibroblasts, smooth muscle cells, endothelial cells and a subset of non-differentiated mesodermal cells. Interestingly, the adherent cellular fraction is typically depleted in many studies by pre-plating cells for the purpose of yielding cardiomyocyte-enriched cultures [19]. However, our studies described below reveal the adherent fraction to be a source of cells capable of differentiation into a cardiomyogenic phenotype.

**Figure 2:**
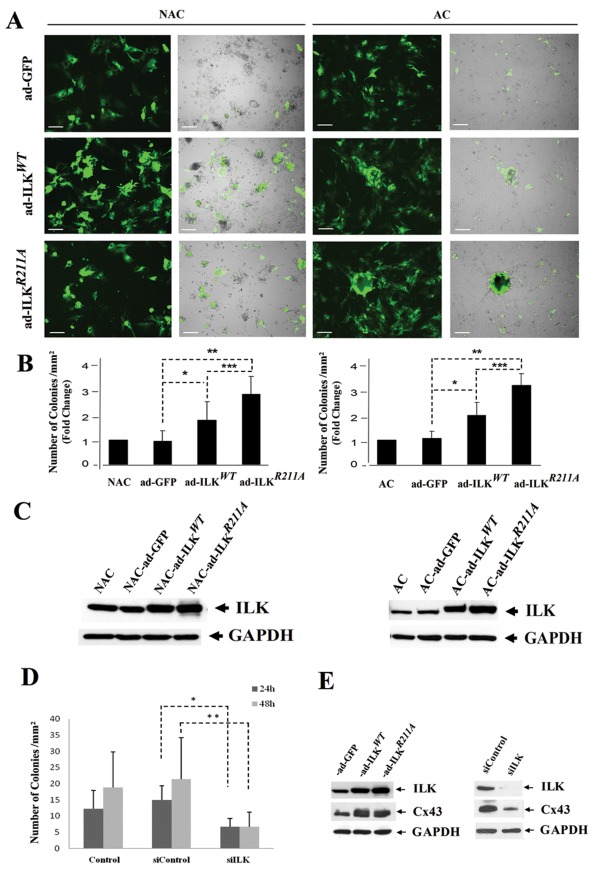
Over-expression of ILK induces robust cellular aggregation. (A) Fluorescent microscopy images and their composites with the phase contrast images identify the cells transduced with GFP-linked vectors (ad-GFP), wild type ILK vector (ad-ILK*^WT^*) and mutant ILK vector (ad-ILK*^R211A^*). Scale bar, 80 µm. (B) Quantification of cell aggregates in AC (right panel) and NAC (left panel) infected with ad-ILK*^WT^*, ad-ILK*^R211A^*, ad-GFP and non-infected cells. Bar graphs represent mean values ±SD, n = 10 (random fields), *p<0.02, **p<0.02, ***p<0.03 (for AC) and *p<0.001, **p<0.0001, ***p<0.001 (for NAC). Note that ad-ILKWT and ad-ILKR211A are significantly different from ad-GFP and that ad-ILKR211A is significant different from ad-ILKWT. (C) Western-blot analysis demonstrates a progressive increase in the level of ILK expression in AC and NAC transduced with ad-ILK*^WT^*, ad-ILK*^R211A^* as compared to non-transduced cells and cells transduced with the vector bearing the GFP-encoding message only. GAPDH was used as a loading control. (D) The number of aggregates was enumerated in human fetal cardiac cells cultures treated with ILK siRNA or scrambled siRNA. 2-tailed p<0.05 at 24 (*) and 48 (**) hours post-transduction. (E) ILK (WT and R211A) overexpression increases the coexpression of ILK and C×43 (left panel); ILK siRNA reduces the endogenous expression levels of these proteins (right panel).

To determine the effects of ILK overexpression on cardiomyogenic morphogenesis, both adherent and non-adherent cells were transduced with adenovirus encoding a cDNA for either human wild type ILK-GFP (ad-ILK*^WT^*) or empty adenoviral GFP control (ad-GFP). We found, that 4 days after transduction, ∼80% of cells expressed GFP in both adherent and initially non-adherent fractions of myocardium-derived cells indicating high efficiency of transduction (Figure 2A). The ad-ILK*^WT^* transduced cultures yielded numerous spherical aggregates, representing about 2 fold increase compared to non-transduced control cultures and to cultures transduced with the empty vector alone (p<0.001) (Figure 2B). Moreover, ad-ILK*^WT^* induced aggregates were comprised of GFP positive cells, whereas the sparse aggregates in the control groups did not show conspicuous GFP staining. The increased levels of ILK protein expression in ad-ILK*^WT^* cultures were confirmed by Western blot analysis (Figure 2C).

### ILK Overexpression Bypasses the Requirement for Exogenous Growth Factor-mediated PI3K Activation in the Induction of Cardiomyogenesis

To test the requirement for PI3K activation in the ILK-mediated cardiomyogenic effect, we also employed overexpression of a mutant ILK gene deficient in PIP3 binding as a result of a point mutation in its PH domain (ILK*^R211A^*) [4], [20]. ILK*^R211A^* treated cultures demonstrated higher levels of ILK protein expression (∼3-fold increase) as compared to ad-ILK*^WT^* infected cultures (Figure 2C). Ad-ILK*^R211A^* treatment also resulted in significant increase in the number of cellular aggregates, detected in both the initially adherent as well as non-adherent cardiac cell populations (Figure 2B). The finding that the cardiomyogenic effects of WT ILK overexpression were evident in non-adherent and adherent fractions, and the corresponding enhanced response to the ILK R211A mutation (p<0.03, ILK*^WT^ vs.* ILK*^R211A^* (NAC); p<0.001, ILK*^WT^ vs.* ILK*^R211A^* (AC)), indicate that exogenously delivered ILK (WT and R211A) acts cell-autonomously to bypass the requirement for PI3K-dependent activation to transduce survival and differentiation signals.

### Endogenous ILK Enhances Cardioblast Formation

Whereas ILK gain-of-function increased the number of cardioblast-containing aggregates, ILK-targeted siRNA caused a reduction in endogenous ILK expression and a significant decrease in cardioblast aggregate formation after 24 and 48 h (Figure 2D). The reduction in ILK expression resulted in a parallel reduction in the expression of the cardiomyocyte gap junction protein C×43 (Figure 2E). These results suggest that endogenous ILK expression plays an important role in the promotion of human fetal cardiomyogenesis.

### Cardioblasts are Electrophysiologically Immature

**Figure 3:**
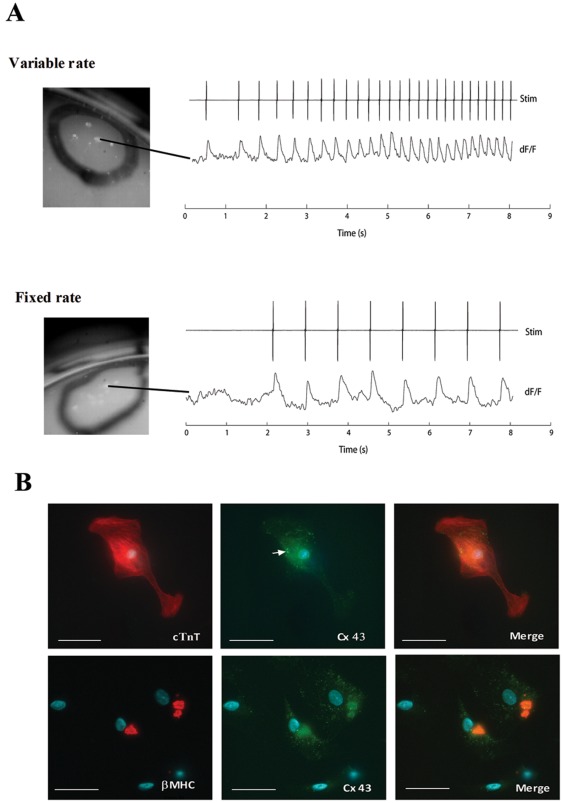
Response of cardioblast aggregates to electrical stimulation. (A) Upper panel shows electrical activity elicited by an increasing rate field electrical stimulation. Lower panel shows another aggregate subject to a constant-rate electrical stimulation. Optical voltage mapping was performed using fluorophore DI-4-ANEPPS as described in Methods. (B) Cells were stained with flouresence-labelled antibodies to β-MHC, C×43 and cTNT. Scale bar, 30 µm.

Optical voltage mapping was performed to determine the response of isolated cardioblast (in aggregates) to electrical field stimulation. Only a small subset (∼5%) of aggregates exhibited electrical responses that were phase-locked to the stimulation train (Figure 3A). Transfection of cells with either ad-GFP, ad-ILK*^WT^* or ad-ILK*^R211A^* did not increase the fraction of aggregates exhibiting electrical response. Although most cardioblasts express β-MHC and the cardiomyocyte gap junction protein C×43, only a small subset of constituent cells expressed the late sarcomeric protein cTnT (Figure 3B), reflecting the relative immaturity of cardioblasts contained in the aggregates, and providing a possible explanation for the electrically quiescent phenotype observed.

### Characterization of ILK-induced Cardioblasts

**Figure 4:**
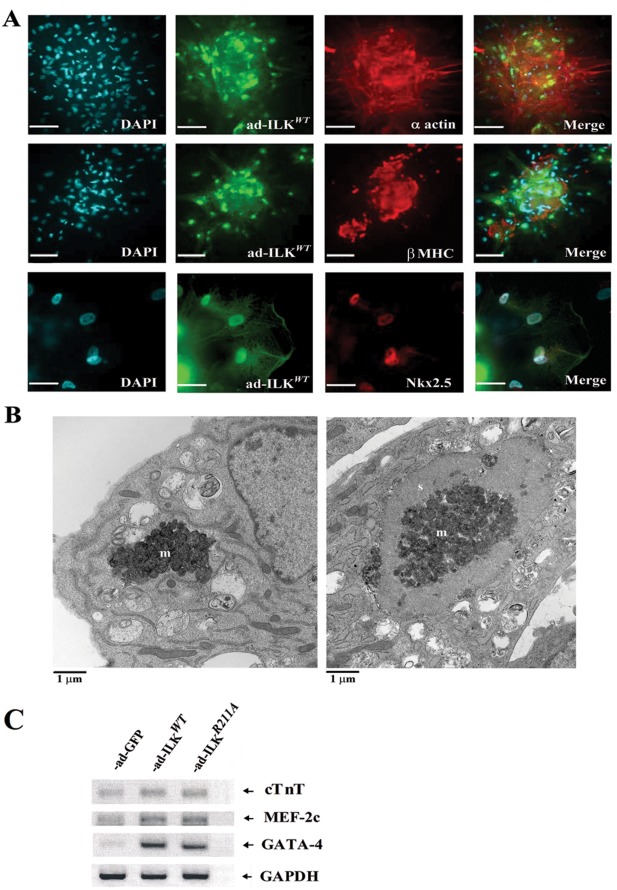
Cellular aggregates in ILK over-expressing cultures are mostly comprised by cardiooblasts. (A) Immunocytochemistry indicates that cellular aggregates present in the ILK*^WT^* -transduced cultures contain numerous cardioblasts displaying the presence of α-actin, β-MHC and nk×2.5 (all marked with red rhodamine). Nuclei were identified with blue DAPI and expression of ILK was marked with green GFP. In top and middle panels, scale bars represent 80 µm; in the bottom panel, scale bar represents 25 µm. (B) Transmission electron microscopy showing clusters of mitochondria (m) in the cytoplasm of the primitive cardioblast (left). The more differentiated cells (right panel) contained similar mitochondrial clusters located in close proximity to the nascent sarcomeres (s). (C) Cardioblasts transduced with ILK (WT and R211A) and control vector were analyzed by RT-PCR for expression of cTNT, GATA-4 and MEF-2c transcripts.

Nk×2.5 is the primordial homeodomain transcription factor required for cardiac gene expression and is specifically essential for left ventricular development [21]. To further characterize the content of cell aggregates in response to ILK upregulation, we probed our cell cultures with antibodies to the early cardiac lineage marker nk×2.5, the cardiomyocyte markers α-actin and sarcomeric protein β-MHC and to α-SMA, a smooth muscle actin-specific marker. We have established that aggregates induced by ad-ILK*^WT^* (Figure 4A) prevalently consisted of cells demonstrating the presence of cardioblast marker nk×2.5 and β-MHC. The same cellular features were also observed in aggregates induced by ad-ILK*^R211A^* and even those sparse aggregates in ad-GFP and non-infected control cells. EM of constituent cells revealed sarcomeric structures of variable size and degrees of organization (Figure 4B). These aggregates also contained scattered endothelial cells and other α-SMA-negative cells that did not display any GFP and thus were non ILK-transduced cells (data not shown). The cardiomyogenic effects of ILK (WT and R211A) were further evident by the increased expression of cardiomyogenic transcription factors MEF-2C and GATA-4 as determined by reverse transcriptase RT-PCR (Figure 4C).

### ILK Co-localizes with Sarcomeric β-MHC

**Figure 5:**
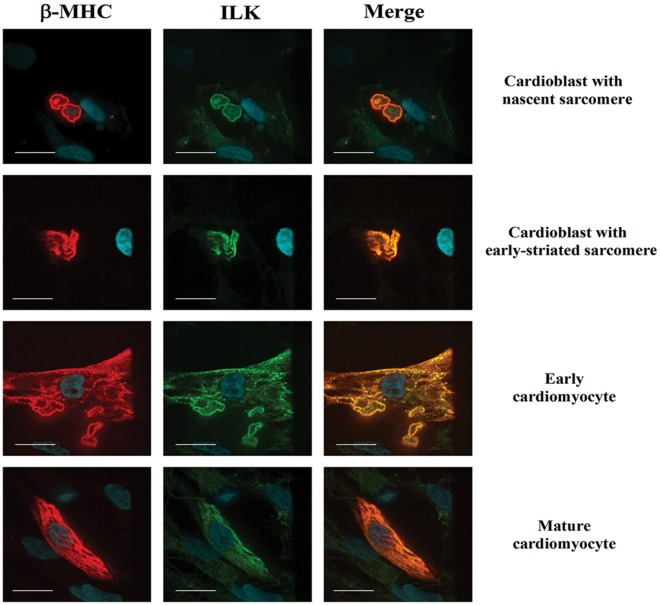
ILK co-localizes with β-MHC in sarcomeres of fetal cardiac cells. Immunocytochemistry of primary cultures of fetal myocardium-derived cells indicates that ILK expression is present in cells representing all stages of cardioblastic-cardiomyogenic differentiation. Confocal microscopy showing human fetal heart derived cells (22 weeks gestation) cultured for 2 days and immunostained with anti-β-MHC (MF-20) (red) and anti-ILK (green) antibodies. Nuclei were detected with DAPI staining (blue). Scale bar, 10 µm.

Confocal microscopy of primary cultures of fetal heart-derived cells immunostained with anti-ß-MHC and anti-ILK antibodies revealed that both detected antigens invariably overlap in the cytoplasm of cells displaying different stages of cardiomyogenic differentiation (Figure 5). In non-treated control cultures most of the β-MHC-positive cells were early cardioblasts exhibiting nascent sarcomeric structures. Only occasional cells represented fully differentiated cardiomyocytes containing well-developed striated sarcomeres. The co-localization of ILK with sarcomeric β-MHC throughout progressive stages of human cardiomyocyte differentiation implies a role for ILK in the morphogenesis of functional sarcomeres.

**Figure 6:**
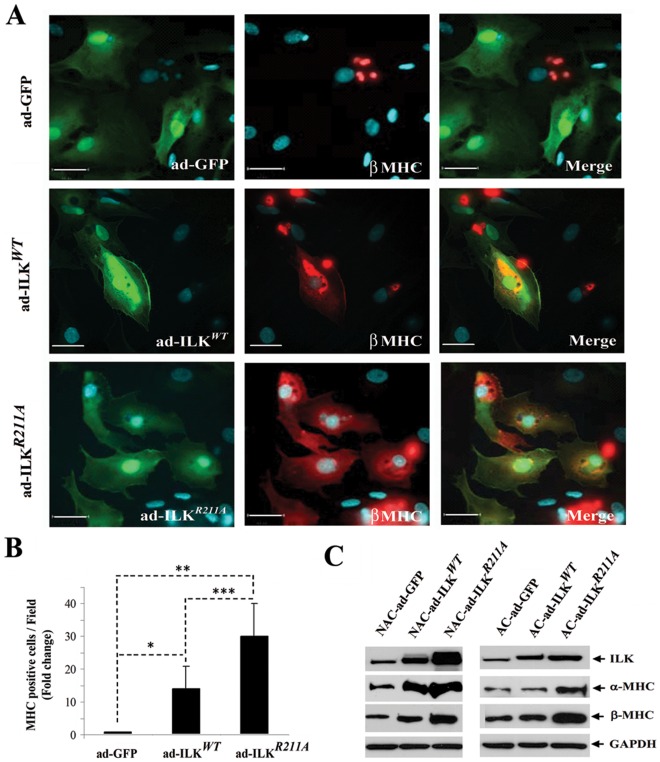
Over-expression of ILK in human fetal cardiac cells induces production of β-MHC. (A) Immunofluorescent staining for β-MHC (red) in human fetal heart-derived cells (21 weeks gestation) infected with adenovirus encoding ad-ILK*^WT^*, ad-ILK*^R211A^* and ad-GFP. Nuclei were detected with DAPI staining (blue). Scale bar, 30 µm. (B) Quantification of the number of β-MHC positive cells detected by immunostaining in adherent fetal cardiomyocytes infected with adenovirus encoding ad-ILK*^WT^*, ad-ILK*^R211A^* and ad-GFP. Bar graphs represent mean values ± SD, n = 14 (random fields), *p<0.02, **p<0.001, ***p<0.03. (C) Western Blot analysis for ILK, cardiac specific α-MHC and β-MHC expression levels in adherent (AC) and non-adherent (NAC) fetal cardiac fractions infected with ad-ILK*^WT^*, ad-ILK*^R211A^* and ad-GFP. Each experiment was performed at least three times on independent samples and one representative blot is shown.

Further analysis of cells transduced with ad-ILK*^WT^* or ILK*^R211A^* showed that increased expression of ILK in ad-ILK*^WT^* or ad-ILK*^R211A^* cultures coincided with a proportional increase in the number of β-MHC-positive cells (WT, p<0.021; R211A, p<0.001) (Figure 6A and 6B) and with the total amount of α- and β-MHC protein detected by Western blot analysis (Figure 6C), consistent with a dosage-dependent sarcomerogenic effect of ILK.

### Overexpression of ILK Stimulates the Expression of *Isl1 in vitro* and *in vivo*

The LIM-homeodomain transcription factor *Isl1* demarcates a distinct cardiac lineage referred to as the second heart field [22], and has been implicated as part of an early transcriptional network which commits a cardiac fate in mesodermal cells [23], [24]. Loss-of-function studies have confirmed the requirement for the expansion and survival of *Isl1* cardiac precursors in mouse embryonic and human neonatal hearts *in vivo* [25], [26].

**Figure 7:**
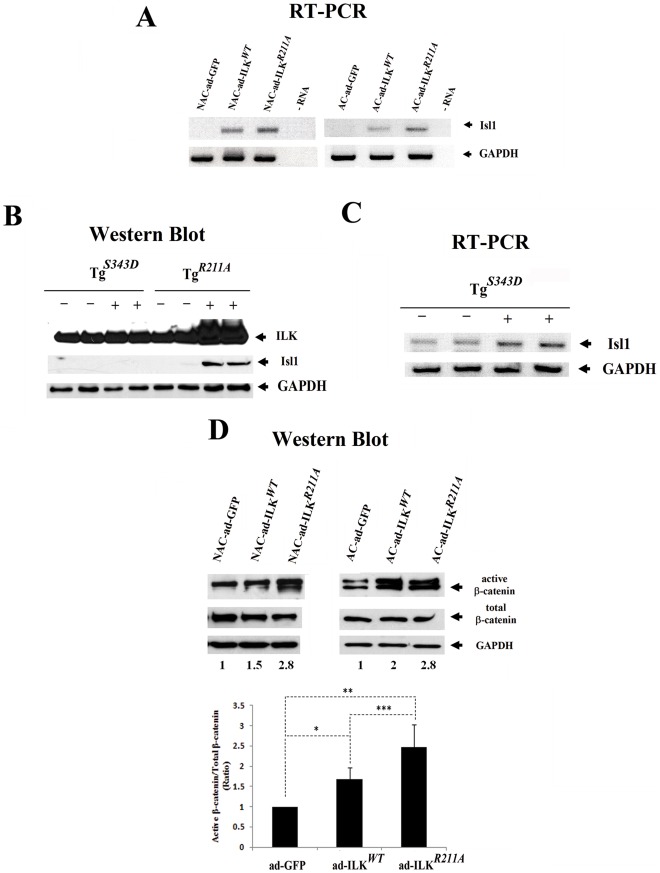
Over-expression of ILK induces *Isl1* expression and β-catenin stabilization *in vitro* and *in vivo.* (A) Semi-quantitative RT-PCR analysis showing the *Isl1* expression in adherent (AC) and non-adherent (NAC) cells derived from fetal myocardium transduced with ad-ILK*^WT^*, ad-ILK*^R211A^* or ad-GFP. GAPDH expression was also tested in all experimental groups. (B) Western Blot analysis of protein levels of ILK and *Isl1* in myocardial lysates derived from transgenic mice with cardiac-restricted expression of constitutively active ILK (Tg*^S343D^*) or mutant ILK (Tg*^R211A^*) and their littermate controls. (C) Semi-quantitative RT-PCR analysis demonstrating the *Isl1* expression in hearts of transgenic mice with cardiac-restricted expression of constitutively active ILK (Tg*^S343D^*) (+) compared to their littermate controls (−). (D) Western blot analysis showing the protein levels of stabilized, dephosphorylated β-catenin and total amount of β-catenin in adherent and non adherent fetal cardiac fractions infected with ad-ILK*^WT^*, ad-ILK*^R211A^* or ad-GFP. Each experiment was performed at least three times on independent samples and one representative blot with its corresponding expression ratios (active/total β-catenin expression) is shown at the top. The bar graph at the bottom summarizes the quantitative analysis of stabilized β-catenin expression in cells derived from fetal myocardium transduced with ad-ILK*^WT^*, ad-ILK*^R211A^* or ad-GFP. Data are mean ± SD, *p<0.007, **p<0.001, ***p<0.05.

While ILK treatment increases the number of cardioblasts *in vitro*, it is remains unclear whether ILK specifies a cardiomyocyte fate among uncommitted mesodermal cells, or whether it induces proliferation of existing cardioblasts. To distinguish these possibilities, *Isl1* expression was determined using RT-PCR in both adherent and non-adherent human fetal cellular fractions transduced with ad-ILK*^WT^*, ad-ILK*^R211A^* and ad-GFP control. Results of these experiments revealed the induction of *Isl1* expression only in ad-ILK*^WT^* and ad-ILK*^R211A^* cultures, but not GFP control-treated cells (Figure 7A).

To confirm this finding *in vivo* we assayed the protein levels of *Isl1* by Western blot analysis of ventricular lysates from transgenic mice with cardiac-restricted expression of constitutively active ILK (ILK*^S343D^*) or the PH domain mutant ILK (ILK*^R211A^*) [4]. As demonstrated in Figure 7B, the protein levels of *Isl1* in ILK*^R211A^* transgenic mice were markedly higher than in littermate controls. The expression levels of *Isl1* in the ILK*^S343D^* genotype were undetectable by Western blot; however, RT-PCR analysis revealed higher *Isl1* mRNA expression in the hearts of ILK*^S343D^* than in littermate controls (Figure 7C). These results indicate that the expression levels of ILK positively correlate with those of *Isl1*. Since *Isl1* is also expressed in cardiac progenitor cells (CPCs) [27], we cannot exclude proliferation of CPCs or that of cardioblasts as contributory mechanisms for the generation of the increased ILK-induced *Isl1* signal, and consequently, for the increased frequency of cardioblast aggregates.

### ILK Activates β-catenin in Human Fetal Cardiomyocytes

Upregulation of ILK leads to the activation and nuclear translocation of β-catenin through inhibition of the β-catenin phospho-degradation complex [28], which in turn, binds to myogenic determination genes including β-MHC [29]. As shown in Figure 7D the expression level of the stabilized, dephosphorylated form of ß-catenin was markedly increased in both, ad-ILK*^WT^*- and ad-ILK*^R211A^*-transduced cultures, as compared to control ad-GFP infected cells (p<0.001; ad-ILK*^R211A^* or ad*-* ILK*^WT^ vs.* ad-GFP). Increased expression of activated ß-catenin was apparent in both the adherent and cardiomyocyte-enriched non-adherent cell fractions. Since β-catenin is required for *Isl1* expression in cardiac progenitor cells and directly regulates the *Isl1* promoter [25], our results support a model in which ILK initiates the early cardioblastic commitment of mesodermal precursor cells through a signaling pathway minimally involving ILK, β-catenin and *Isl1*.

### ILK is Expressed During Cardiogenesis in Human Embryonic Stem Cells

**Figure 8:**
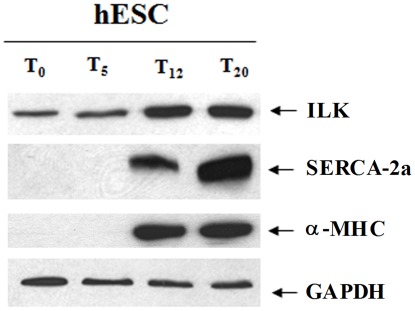
Endogenous ILK is expressed during human embryonic stem cell (hESC) cardiogenesis. The induction of ILK is coincident with increasing expression of cardiomyocyte-specific sarcoplasmic endoplasmic reticulum calcium ATPase, isoform 2a (SERCA2a) and α-MHC. T_0−20_ marks the time in days during differentiation of cells from embryoid bodies to cardiomyocytes.

Increasing expression of endogenous ILK level was also confirmed during growth factor-induced early cardiogenesis using an optimized combination of Activin A, Nodal and Bone Morphogenic Protein-4 in human embryonic stem cells (hESC) (Figure 8) [30]. Here, ILK expression coincided with that of the cardiac isoform of sarco(endo)plasmic reticulum calcium ATPase (SERCA2a) and α-MHC, indicating that endogenous ILK is upregulated during induction of the cardiomyogenic program in hESCs.

## Discussion

Data presented in this study indicate for the first time that experimental overexpression of the multifunctional serine/threonine kinase ILK promotes, in vitro, recruitment of human fetal heart cells to a cardiomyogenic fate. ILK gain-of-function is shown to induce the differentiation of a resident mesodermal precursor cell toward an early cardiomyocyte characterized by nascent sarcomeric structures, and to lead to subsequent cardiomyogenic maturation through increased sarcomerogenesis. Proliferation and cellular hypertrophy of existing cardioblasts have been inferred as the mechanisms accounting for somatic growth of the fetal heart at a stage after septation [31], [32]. However, our finding that accelerated cardiomyocyte specification is inducible by overexpression of ILK at a stage subsequent to definitive ventricular chamber formation suggests the persistence of a recruitable precursor cell population late in human cardiac development. Further studies are required to identify the specific precursor cells that give rise to cardioblasts in response to ILK overexpression. In further support of the cardiomyogenic role of endogenous ILK, we demonstrate that targeted reduction of ILK expression using siRNA led to a reduction in cardioblast formation. Our studies also demonstrate that expression of endogenous ILK progressively increases during growth factor-induced early cardiogenesis in hESCs. Support for a critical role of ILK in the initiation and maintenance of the normal cardiomyogenic phenotype is also found in previous studies showing that disruption of ILK signaling during development leads to neonatal cardiomyopathy [9], [10].

Most studies of the signaling pathways during heart development concern those regulating secondary heart field (SHF) development, which forms the definitive outflow tract, including those regulated by Wnt, fibroblast growth factor, bone morphogenetic protein, Hedgehog, and retinoic acid signaling (reviewed in [33], whereas more limited data is available concerning pathways responsible for the primary or first heart field (FHF), which comprises the dominant portion of the left ventricle. The results of our study of ILK-induced cardiomyogenesis implicate a more global role for β-catenin activation in ventricular morphogenesis, and are consistent with previously published results showing that activation of Wnt/β-catenin pathway enhances embryonic stem cell differentiation into cardiomyocytes [17], accelerates cardiogenesis in the undifferentiated P19CL6 cell line [34], and promotes the expansion of cardiac progenitor cells and is required for cardiac differentiation [35].

Other studies indicate more complex context-specific and antagonistic effects of Wnt signaling on cardiomyogensis [14], [17]. ILK can also signal through a c-Jun-N-terminal kinase (JNK/c-Jun) signaling axis independently of the canonical signaling target GSK-3β [36]. Since non-canonical Wnt signaling via Wnt11 is sufficient to induce cardiomyogenesis in bone marrow mononuclear cells in a JNK/c-Jun-dependent manner [37], the myogenic effects of ILK may depend upon the contextual balance of canonical and non-canonical Wnt signaling. It was previously shown that ILK elevates the protein translation mediator P70S6 kinase (p70S6K) during cardiac hypertrophy [4], and independently that p70S6K induces differentiation in human embryonic stem cells [38]. The precise molecular mechanism of the effect of ILK overexpression on induction of cardioblast in human fetal heart remains to be determined.

Cardioblasts generated by ILK overexpression displayed low-level organization of sacromeric structures and were nk×2.5-positive indicative of a cardiomyogenic lineage. Interestingly, a population of early presumptive cardioblasts in the adherent fraction displayed coexistence of intracellular collagen fibrils and early sarcomeres, possibly indicating their origin from progenitors with both fibroblastic and cardiomyogenic potential. Cells exhibiting progressively more distinct striations typical of more differentiated cardiomyocytes were mostly present in non-adherent aggregates and stained positively predominantly for β-MHC representing the fetal isoform of sarcomeric protein. Moreover, confocal microscopy revealed localization of ILK to sarcomeres suggesting a possible role for ILK in nucleating new sarcomeres, and providing a basis for the pro-hypertrophic effects observed with ILK activation [4], [6]. Co-localization of ILK and sarcomeres was also previously reported in fully differentiated cardiomyocytes in zebrafish [9].

ILK overexpression was shown here to increase the expression of the early marker of cardiogenesis, *Isl1*, in human cardiac cells *in vitro* and in the ILK transgenic mouse lines. Previous studies have suggested that *Isl1* may also be expressed in FHF and thus represent a pan-cardiocytic marker for both myocardial cell lineages [27], [33] and may account for the finding herein of ILK-induced *Isl1* expression in human fetal cardiac cells that are assumed to derive predominantly from the FHF-derived ventricular mass. Our previous study demonstrated an increase in the c-Kit progenitor cell compartment in ILK-treated human fetal cell-derived cultures [39]. The current study does not address the nature of specific cardiac precursor cells that are responsive to ILK induction, which is better approached by examination of the effects of defined factors on cellular phenotypes present at pre-cardiomyogenic stages using a hESC platform [30]. A recent advance toward this objective was the identification of signal regulatory protein alpha (SIRPA) as a cardiomyocyte-specific marker that distinguishes the cardiomyogenic lineage from nonmyocyte precursor cells among populations of human induced pluripotent stem cells and hESCs [40].

The majority of cardioblasts examined in our study were not spontaneously contractile, and only a small subset was responsive to electrical stimulation. Previous reports describing a higher proportion of contractile cells were based on subcloning methods to isolate CPCs, which then underwent spontaneous or directed cardiomyogenic differentiation [41], [42]. Our studies, in contrast, investigated the phenotype of unselected populations of cells that were not enriched for progenitor cells and contained cardiomyocytes reflecting the spectrum of differentiation likely to be present *in vivo*. The relative electrical quiescence of freshly isolated human fetal and adult cardiac cells has led to the use of iPSC [43] - and hESC [30] -derived cardiogenesis as more robust models to investigate the electrophysiological properties of human cardiomyocytes.

Altogether, our results identify ILK as a novel regulator in the induction of cardiomyogenic fate. Remarkably, ILK may serve a dual function, regulation of sequential phases of cardiomyogenesis, and, as a mechanoreceptor protein, stress-dependent modulation of cardiomyocyte contractility throughout postnatal life [4], [5]. These diversified functions of ILK are unified by the process of sarcomeric morphogenesis which is responsive to mechanical stress during development and during dynamic mechanical loading postnatally. The multifunctional properties of ILK contrast with that of orthodox cardiomyogenic transcription factors, such as *Isl1* and nk×2.5, which are activated transiently during cardiogenesis. The positioning of ILK as a single upstream node but with both structural and signal transduction properties leveraged by extensive protein-protein interactions [5], [44], represents an efficient molecular mechanism to orchestrate multiple aspects of cardiomyocyte development and function.

Interestingly, overpression of the activation resistant ILK R211A mutant resulted in much greater increase in the number of new cell aggregates as compared to overexpression of wild-type ILKWT. The potency of the cardiomyogenic effects of the ILK R211A mutation are consistent with a model in which ILK (WT and R211A) exogenously delivered to the cell bypasses the requirement for proximal PI3K-dependent activation. The ILK R211A mutation possesses a normal kinase domain and thus retains signaling competence, and exhibits high expression levels, and these factors may account for its cardiomyogenic potency. This feature may have translational significance since the R211A mutation appears to be phenotypically inert and thus potentially less oncogenic [45] than other prosurvival molecules or stem cell based approaches.

Since ILK is known to be activated by hypoxia, which normally occurs during fetal heart development [46] and in postnatal heart disease [4], these findings support the paradigm that stress induction of ILK might serve as a novel endogenous regulator of cardiomyogenesis. The identification of ILK as a nodal regulatory element during cardiomyogenesis increases the scope of potential translational approaches designed to exploit this pathway. Furthermore, our data encourages future studies aimed at controlled activation of ILK pathway that may be useful in promoting the cardiomyogenic differentiation of induced pluripotent stem cells, and may eventually lead to the development of novel therapeutic approaches allowing regeneration of diseased human myocardium.

## Material and Methods

### Cell Isolation and Culture

Human fetal hearts were harvested following elective pregnancy termination at 19 to 22 weeks gestation. Approval by the Human Research Ethics Board of the Hospital for Sick Children and written maternal consent were obtained for this study. The hearts were minced and washed with phosphate-buffered saline. Cells isolation was performed with 0.2% trypsin and 1 mg/ml type II collagenase in 0.02% glucose phosphate-buffered saline (PBS), pH7.4 solution at 37°C. After dissection, cells were incubated on plastic culture dishes (Sarstedt, Inc, Newton, NC) for 2 hours at 37°C to separate cells for adherent and non-adherent cells, with Iscove’s modified Dulbecco’s medium (IMDM, Gibco, Invitrogen Corporation, Carlsbad, Calif) containing penicillin and streptomycin and supplemented with 10% fetal bovine serum (FBS, Gibco). After incubation, the supernatant with non-adherent cells was transferred to new culture dishes (Sarstedt). Both adherent and non-adherent cells were placed in a 5% carbon dioxide incubator at 37°C prior to infection.

### *In vitro* Studies Using ILK Adenoviral Infection

Cells were cultured to 60%–70% confluency prior to adenovirally mediated infection with serotype 5 adenovirus encoding either human wild-type ILK gene (ad-ILK*^WT^*) or a mutant ILK gene (ad-ILK*^R211A^*) in a bicistronic construct containing GFP as a reporter gene [4]. Control cultured cells were infected with adenovirus containing the reporter gene alone (ad-GFP). Cells were infected at 37°C at multiplicity of infection of 1.5 in IMDM media supplemented with 10% FBS for 24 hours and analyzed 3–5 days after infection. The infection efficiency was confirmed by the expression of GFP.

### Western Blots

For western blot analysis, total and phospho-specific protein expression was measured in lysates derived from human fetal cardiomyocytes in culture and from transgenic and control mouse ventricular tissue as described previously [4]. Briefly, cells extracts were prepared by lysing cells for 20 min on ice in RIPA lysis buffer (150 mM NaCl, 1% Nonidet P40, 05% deoxycholate, 0.1% SDS, 50 mM Tris, pH 8.0, and 1 mM PMSF). The expression levels of proteins was assessed using the following primary antibodies: rabbit monoclonal anti-ILK (Clone 4G9, Cell Signaling Technologies), rabbit polyclonal anti-Isl1 (Chemicon International, Inc), mouse monoclonal anti-β-catenin (Clone E-5, Santa Cruz Biotechnology, Inc), mouse monoclonal anti-active-β-catenin (Clone 8E7, Millipore), monoclonal anti-GAPDH (Clone GAPDH-71.1, Sigma), mouse monoclonal anti-myosin heavy chain-β (Clone A4.951, Santa Cruz Biotechnology, Inc), rabbit polyclonal anti-α myosin heavy chain (Sigma). After incubation with the primary antibody, the blots were washed and incubated for 1 h with the appropriate horseradish peroxidase-conjugated secondary antibody (Jackson ImmunoResearch Laboratories). Proteins were visualized with an enhanced chemiluminescence (ECL) detection reagent (Amersham Pharmacia Biotech) and quantified by densitometry.

### Immunohistochemical Analysis

Cultured cells on coverslips were maintained in culture for 4–7 days and then fixed with 4% paraformaldehyde for 20 min at room temperature. Following rinsing with PBS, cells were permeabilized with 0.1% Triton-X 100 (Sigma) for 10 min and then blocked with normal goat serum or 5% milk for 30 min and subjected to immunostaining. The following primary antibodies were used in this study: rabbit polyclonal anti-vimentin (Abcam Inc), rabbit monoclonal anti-ILK (Clone 4G9, Cell Signaling Technologies), mouse monoclonal anti myosin heavy chain-b (Clone MF-20, provided by Dr. Donald A. Fischman, Cornell University Medical College, NY), mouse monoclonal anti-nk×2.5 (Clone 259416, R&D Systems), mouse monoclonal anti α-actin (Clone 1A4, Santa Cruz Biotechnology, Inc), rabbit polyclonal anti-ki-67 (Millipore), mouse monoclonal anti cardiac Troponin T (Clone 13–11, Thermo Fisher Scientific), rabbit polyclonal anti-connexin 43 (Sigma). Nuclei were stained with 4,6-diamino-2-phenylindole (DAPI). All analysis was done with OpenLab 4.0.2 software (Agilent Technologies, Scientific Software Inc, Palo Alto, Ca.).

### Semi-quantitative Reverse Transcription (RT–PCR)

Total RNA from cultured cells or heart tissue was prepared by using TRIzol® (Invitrogen) according to the manufacturer’s instructions. Total RNA (1 µg) was reverse-transcribed with the SuperScript First-strand Synthesis System (Invitrogen). The cDNA of targeted genes was then amplified using the following primers: mouse *Isl1*: 5′-CGTGCAGACCACGATGTGG-3′ and 5′-GACTGAGGCCCGTCATCTC-3′; human *Isl1*: 5′- CACGATCAGTATATTCTGAG-3′ and 5′-CGTGGTCTGCTCGGCAGAAG-3′; human cTnT: 5′ –ATGAGCGGGAGAAGGAGCGG CAGAAC-3′ and 5′ -TCAATGGCCAGCACCTTCCTCCTCTC-3′; human MEF-2c: 5′ –TTTA ACACCGCCAGCGCTCTTCACCTTG-3′ and 5′ –TCGTGGCGCGTGTGTTGTGGGTATCT CG-3′; human GATA-4: 5′ –GCTCGTGCGCCACCTCCAGGC-3′ and 5′ –GGCAAC AACGA TAATATGCG-3′. A total of 30 amplification cycles were performed. To control for the amount of intact RNA, GAPDH was amplified in parallel with the following primers: mouse GAPDH: 5′-AGGGCTGCCATTTGCAGTGG-3′ and 5′- CATTTGATGTTAGTGGGGTCT -3′; and human GAPDH: 5′- CGGATTTGGTCGTATTGGGC-3′ and 5′- CTCCATGGTGGTGAAG ACG-3′.

### RNA Interference, Transfection, and Transduction

siRNA against ILK (siILK) and negative control siRNA were purchased from Cell Signaling Technologies. Cells were transfected with siRNAs (130 µM final concentration) using the X-tremeGENE siRNA transfection reagent (Roche Diagnostics, Laval, QC, Canada) according to the manufacturer’s instructions. To induce transduction efficiency the cells were transfected again after 24 hours with the same concentration of siRNA. The cells were analysed after 48 h post-transfection.

### Transmission Electron Microscopy (EM)

For electron microscopic analysis, cells were fixed for 10 min in 1% glutaraldehyde 4% formaldehyde mixture in PBS, scraped off and pelleted. Fixation was continued for 1 h. After fixation, the cells were post-fixed in 1% solution of osmium tetroxide and dehydrated in graded acetone at 4°C. After embedding and polymerization, 0.5-µm-thick sections were initially cut with a Leica Ultracut UCT ultramicrotome, stained in uranyl acetate and lead citrate, and observed with transmission electron microscopy at 80 kV using a Philips CM100 transmission electron microscope.

### Generation of Transgenic Mouse Lines

The methods for generation of transgenic animals conveying cardiac-specific over-expression of human ILK gene containing a PH-domain (ILK*^R211A^*) and constitutive-active (ILK*^S343D^*) mutation have been previously described [4].

### Generation of Human Embryonic Stem Cells

hESCs were maintained in defined culture and differentiated into cardiomyocytes using stage-specific exposure to nodal, activin and BMP, as described [30].

### Electrical Responsiveness of Human Fetal Cardiomyocytes

Optical voltage mapping was performed using fluorophore DI-4-ANEPPS. The cell cultures were incubated with 5 uM DI-4-ANEPPS for 20 minutes. Optical mapping was performed using a high-speed camera (SciMedia USA) equipped with a 100×100 pixel CMOS sensor. The fluorescence was excited using a 150 W halogen lamp (Moritek Corp Japan) with a 530 nm bandpass filter and was measured using a microscope and a 610 nm long pass filter (THT, SciMedia USA) which provided a 5 mm×5 mm field of view.

### Statistical Analysis

Statistical comparison of ILK-specific effects relied on a paired *t* test or analysis of variance (ANOVA) followed by the multiple-comparison Bonferroni *t* test to assess differences among groups. The significance level was set at p<0.05.

## References

[1] Legate KR, Wickstrom SA, Fassler R (2009). Genetic and cell biological analysis of integrin outside-in signaling.. Genes Dev.

[2] Legate KR, Fassler R (2009). Mechanisms that regulate adaptor binding to beta-integrin cytoplasmic tails.. J Cell Sci.

[3] Hannigan G, Troussard AA, Dedhar S (2005). Integrin-linked kinase: a cancer therapeutic target unique among its ILK.. Nat Rev Cancer.

[4] Lu H, Fedak PW, Dai X, Du C, Zhou YQ (2006). Integrin-linked kinase expression is elevated in human cardiac hypertrophy and induces hypertrophy in transgenic mice.. Circulation.

[5] Hannigan GE, Coles JG, Dedhar S (2007). Integrin-linked kinase at the heart of cardiac contractility, repair, and disease.. Circ Res.

[6] Xie J, Lu W, Gu R, Dai Q, Zong B (2011). The Impairment of ILK Related Angiogenesis Involved in Cardiac Maladaptation after Infarction.. PLoS ONE.

[7] Sakai T, Li S, Docheva D, Grashoff C, Sakai K (2003). Integrin-linked kinase (ILK) is required for polarizing the epiblast, cell adhesion, and controlling actin accumulation.. Genes Dev.

[8] Samarel AM (2005). Costameres, focal adhesions, and cardiomyocyte mechanotransduction.. Am J Physiol Heart Circ Physiol.

[9] Bendig G, Grimmler M, Huttner IG, Wessels G, Dahme T (2006). Integrin-linked kinase, a novel component of the cardiac mechanical stretch sensor, controls contractility in the zebrafish heart.. Genes Dev.

[10] White DE, Coutu P, Shi YF, Tardif JC, Nattel S (2006). Targeted ablation of ILK from the murine heart results in dilated cardiomyopathy and spontaneous heart failure.. Genes Dev.

[11] Delcommenne M, Tan C, Gray V, Rue L, Woodgett J (1998). Phosphoinositide-3-OH kinase-dependent regulation of glycogen synthase kinase 3 and protein kinase B/AKT by the integrin-linked kinase.. Proc Natl Acad Sci U S A.

[12] Abboud ER, Coffelt SB, Figueroa YG, Zwezdaryk KJ, Nelson AB (2007). Integrin-linked kinase: a hypoxia-induced anti-apoptotic factor exploited by cancer cells.. Int J Oncol.

[13] Lee SP, Youn SW, Cho HJ, Li L, Kim TY (2006). Integrin-linked kinase, a hypoxia-responsive molecule, controls postnatal vasculogenesis by recruitment of endothelial progenitor cells to ischemic tissue.. Circulation.

[14] Qyang Y, Martin-Puig S, Chiravuri M, Chen S, Xu H (2007). The renewal and differentiation of Isl1+ cardiovascular progenitors are controlled by a Wnt/beta-catenin pathway.. Cell Stem Cell.

[15] Yi F, Pereira L, Merrill BJ (2008). Tcf3 functions as a steady-state limiter of transcriptional programs of mouse embryonic stem cell self-renewal.. Stem Cells.

[16] Tam WL, Lim CY, Han J, Zhang J, Ang YS (2008). T-cell factor 3 regulates embryonic stem cell pluripotency and self-renewal by the transcriptional control of multiple lineage pathways.. Stem Cells.

[17] Naito AT, Shiojima I, Akazawa H, Hidaka K, Morisaki T (2006). Developmental stage-specific biphasic roles of Wnt/beta-catenin signaling in cardiomyogenesis and hematopoiesis.. Proc Natl Acad Sci U S A.

[18] Cary RB, Klymkowsky MW (1994). Differential organization of desmin and vimentin in muscle is due to differences in their head domains.. J Cell Biol.

[19] Engel FB, Hauck L, Cardoso MC, Leonhardt H, Dietz R (1999). A mammalian myocardial cell-free system to study cell cycle reentry in terminally differentiated cardiomyocytes.. Circ Res.

[20] Huang J, Mahavadi S, Sriwai W, Hu W, Murthy KS (2006). Gi-coupled receptors mediate phosphorylation of CPI-17 and MLC20 via preferential activation of the PI3K/ILK pathway.. Biochem J.

[21] Kirby ML (2007). Cardiac Development..

[22] Buckingham M, Meilhac S, Zaffran S (2005). Building the mammalian heart from two sources of myocardial cells.. Nat Rev Genet.

[23] Brade T, Gessert S, Kuhl M, Pandur P (2007). The amphibian second heart field: Xenopus islet-1 is required for cardiovascular development.. Dev Biol.

[24] Anton R, Kuhl M, Pandur P (2007). A molecular signature for the “master” heart cell.. Bioessays.

[25] Lin L, Cui L, Zhou W, Dufort D, Zhang X (2007). Beta-catenin directly regulates Islet1 expression in cardiovascular progenitors and is required for multiple aspects of cardiogenesis.. Proc Natl Acad Sci U S A.

[26] Genead R, Danielsson C, Wardell E, Kjaeldgaard A, Westgren M (2010). Early first trimester human embryonic cardiac Islet-1 progenitor cells and cardiomyocytes: Immunohistochemical and electrophysiological characterization.. Stem Cell Res.

[27] Laugwitz KL, Moretti A, Caron L, Nakano A, Chien KR (2008). Islet1 cardiovascular progenitors: a single source for heart lineages?. Development.

[28] Oloumi A, McPhee T, Dedhar S (2004). Regulation of E-cadherin expression and beta-catenin/Tcf transcriptional activity by the integrin-linked kinase.. Biochim Biophys Acta.

[29] Pan W, Jia Y, Huang T, Wang J, Tao D (2006). Beta-catenin relieves I-mfa-mediated suppression of LEF-1 in mammalian cells.. J Cell Sci.

[30] Kattman SJ, Witty AD, Gagliardi M, Dubois NC, Niapour M (2011). Stage-specific optimization of activin/nodal and BMP signaling promotes cardiac differentiation of mouse and human pluripotent stem cell lines.. Cell Stem Cell.

[31] Ieda M, Tsuchihashi T, Ivey KN, Ross RS, Hong TT (2009). Cardiac fibroblasts regulate myocardial proliferation through beta1 integrin signaling.. Dev Cell.

[32] Kirby ML (2007). Cardiac Development..

[33] Rochais F, Mesbah K, Kelly RG (2009). Signaling pathways controlling second heart field development.. Circ Res.

[34] Liu Z, Li T, Liu Y, Jia Z, Li Y (2009). WNT signaling promotes Nk×2.5 expression and early cardiomyogenesis via downregulation of Hdac1.. Biochem Biophys Acta.

[35] Kwon C, Arnold J, Hsiao EC, Taketo MM, Conklin BR (2007). Canonical Wnt signaling is a positive regulator of mammalian cardiac progenitors.. Proc Natl Acad Sci U S A.

[36] Durbin AD, Somers GR, Forrester M, Pienkowska M, Hannigan GE (2009). JNK1 determines the oncogenic or tumor-suppressive activity of the integrin-linked kinase in human rhabdomyosarcoma.. J Clin Invest.

[37] Flaherty MP, Abdel-Latif A, Li Q, Hunt G, Ranjan S (2008). Noncanonical Wnt11 signaling is sufficient to induce cardiomyogenic differentiation in unfractionated bone marrow mononuclear cells.. Circulation.

[38] Easley CA B-YA, Redinger CJ, Mich-Basso JD, Oliver SL, McFarland DA (2009). Expression of constitutively active P70 S6K, a protein translation mediator, induces differentiation in pluripotenet human embryonic stem cells; July 8–11, 7th Annual Meeting of the International Society for Stem Cell Research.

[39] Yamabi H, Lu H, Dai X, Lu Y, Hannigan G (2006). Overexpression of integrin-linked kinase induces cardiac stem cell expansion.. J Thorac Cardiovasc Surg.

[40] Dubois NC, Craft AM, Sharma P, Elliott DA, Stanley EG (2011). SIRPA is a specific cell-surface marker for isolating cardiomyocytes derived from human pluripotent stem cells.. Nat Biotechnol.

[41] de Boer TP, van Veen TA, Jonsson MK, Kok BG, Metz CH (2009). Human cardiomyocyte progenitor cell-derived cardiomyocytes display a maturated electrical phenotype.. J Mol Cell Cardiol.

[42] Smits AM, van Vliet P, Metz CH, Korfage T, Sluijter JP (2009). Human cardiomyocyte progenitor cells differentiate into functional mature cardiomyocytes: an in vitro model for studying human cardiac physiology and pathophysiology.. Nat Protoc.

[43] Kattman SJ, Koonce CH, Swanson BJ, Anson BD (2011). Stem cells and their derivatives: a renaissance in cardiovascular translational research.. J Cardiovasc Transl Res.

[44] Lange A, Wickstrom SA, Jakobson M, Zent R, Sainio K (2009). Integrin-linked kinase is an adaptor with essential functions during mouse development.. Nature.

[45] Durbin AD, Pasic I, Wong DK, Hannigan GE, Malkin D (2010). The oncogenic and growth-suppressive functions of the integrin-linked kinase are distinguished by JNK1 expression in human cancer cells.. Cell Cycle.

[46] Fudim M BJ, Houben AP, Wernet P, Buchheiser A, Kogler G (2009). The influence of hypoxia on generation, expansion and differentiation of unrestricted somatic stem cells from human cord blood and bone marrow stromal cells..

